# A Data-Driven Approach for Internal Crack Prediction in Continuous Casting of HSLA Steels Using CTGAN and CatBoost

**DOI:** 10.3390/ma18153599

**Published:** 2025-07-31

**Authors:** Mengying Geng, Haonan Ma, Shuangli Liu, Zhuosuo Zhou, Lei Xing, Yibo Ai, Weidong Zhang

**Affiliations:** 1 National Center for Materials Service Safety, University of Science and Technology Beijing, Beijing 100083, China; gengmy@xs.ustb.edu.cn (M.G.); 2 SINO-PIPELINE International, Beijing 102206, China; haonan.ma@cnpc.com; 3 Hesteel Group Tangsteel Company, Tangshan 063000, China; 4 Southern Marine Science and Engineering Guangdong Laboratory (Zhuhai), Zhuhai 519082, China

**Keywords:** continuous casting, machine learning, CTGAN, data-driven methods, internal cracks

## Abstract

Internal crack defects in high-strength low-alloy (HSLA) steels during continuous casting pose significant challenges to downstream processing and product reliability. However, due to the inherent class imbalance in industrial defect datasets, conventional machine learning models often suffer from poor sensitivity to minority class instances. This study proposes a predictive framework that integrates conditional tabular generative adversarial network (CTGAN) for synthetic minority sample generation and CatBoost for classification. A dataset of 733 process records was collected from a continuous caster, and 25 informative features were selected using mutual information. CTGAN was employed to augment the minority class (crack) samples, achieving a balanced training set. Feature distribution analysis and principal component visualization indicated that the synthetic data effectively preserved the statistical structure of the original minority class. Compared with the other machine learning methods, including KNN, SVM, and MLP, CatBoost achieved the highest metrics, with an accuracy of 0.9239, precision of 0.9041, recall of 0.9018, and F1-score of 0.9022. Results show that CTGAN-based augmentation improves classification performance across all models. These findings highlight the effectiveness of GAN-based augmentation for imbalanced industrial data and validate the CTGAN–CatBoost model as a robust solution for online defect prediction in steel manufacturing.

## 1. Introduction

High-strength low-alloy (HSLA) steels are widely used in automotive, pipeline, and structural components due to their superior strength-to-weight ratio and weldability [1]. In modern steel production, continuous casting has become the predominant method for converting molten steel into solid billets [2]. However, during this process, HSLA steels are prone to internal defects, particularly centerline segregation and internal cracks, that compromise product reliability and downstream processability. These defects compromise downstream processability and mechanical performance, making early and accurate prediction of internal cracks essential for improving product quality and reducing production losses [3,4,5,6,7].

These internal cracks typically result from complex thermomechanical phenomena during solidification, such as non-uniform heat transfer, segregation-induced brittleness, and improper mold or secondary cooling strategies [8,9]. Traditional defect control methods based on rule-based systems or offline metallographic inspection are limited by their reactive nature and lack of scalability [10,11].

To achieve proactive and data-driven quality control, researchers have increasingly explored machine learning techniques for defect prediction. In recent years, with the rise of Industry 4.0 and increased deployment of intelligent sensors, machine learning has emerged as a promising tool for data-driven defect prediction using process parameters collected during casting operations [12,13,14]. Numerous studies have applied machine learning models such as multilayer perceptron (MLP) [15], principal component analysis (PCA) combined with support vector machines (SVMs) [16], and K-nearest neighbors (KNNs) [17] to internal crack prediction. For example, Kong et al. [9] integrated stress–strain modeling with an expert system to build a predictive framework with over 86% industrial deployment accuracy. Liu et al. [18] proposed a GANs-based data augmentation strategy that expanded the electroslag remelting dataset, enabling the GANs–DBN model to achieve a prediction accuracy of 91.8%, precision of 84.2%, recall of 90.5%, and F1-score of 87.2% for D-type inclusion detection. Zou et al. [19] employed PCA and DNNs to classify crack-prone billets, achieving over 92% accuracy. Despite these successes, class imbalance remains a persistent challenge in industrial datasets, as internal cracks occur much less frequently than defect-free samples. This imbalance often leads machine learning classifiers to favor the majority class, resulting in high overall accuracy but reduced sensitivity to the minority (cracked) instances.

To address this, data-level augmentation has been widely adopted as a practical solution [20]. Classical methods such as the synthetic minority oversampling technique [21] generate new minority samples through interpolation between nearby data points. These methods have shown effectiveness in domains such as manufacturing defect prediction, medical diagnosis, and fraud detection [15,22]. However, they rely on local linearity assumptions and may introduce synthetic samples in sparse or outlier-prone regions, potentially reducing classifier robustness. Generative Adversarial Networks (GANs) have been proposed as an alternative for learning complex feature distributions and generating realistic synthetic samples [23]. Originally developed for image data, GAN variants such as CTGAN [24] have been adapted for tabular data, offering mechanisms to capture nonlinear, multimodal distributions and relate conditional variables. In industrial applications, GANs have been successfully applied to surface defect synthesis in hot-rolled steels [25], demonstrating promising performance in visual inspection tasks. However, their application to structured process parameter data, such as those obtained from continuous casting operations, has received limited attention and remains insufficiently explored.

In this study, we investigate the use of CTGAN for minority class augmentation in internal crack prediction during continuous casting of HSLA steels. A real-world dataset comprising 733 samples was collected from an industrial continuous casting machine. Mutual information was employed for feature selection, and the top 25 features were selected based on their mutual information scores with the target label. CTGAN-based augmentation was then employed to balance the dataset. We evaluate the effectiveness of the augmented data through distributional alignment and PCA visualization and compare the performance of the CTGAN–CatBoost framework with other machine learning classifiers, including KNN, SVM, and MLP.

## 2. Data Description and Preprocessing

### 2.1. Data Source and Cleaning

Continuous casting is the most used process for steelmaking due to its efficiency [26,27]. The data used in this study were collected from the No. 1 continuous casting machine at Hesteel Group Tangsteel Company, located in Tangshan, Hebei Province, China. It represents real-world production records from an HSLA steel production line. To facilitate reproducibility and contextual understanding, Table 1 summarizes the main equipment parameters of the industrial caster used in this study.

**Figure 1:**
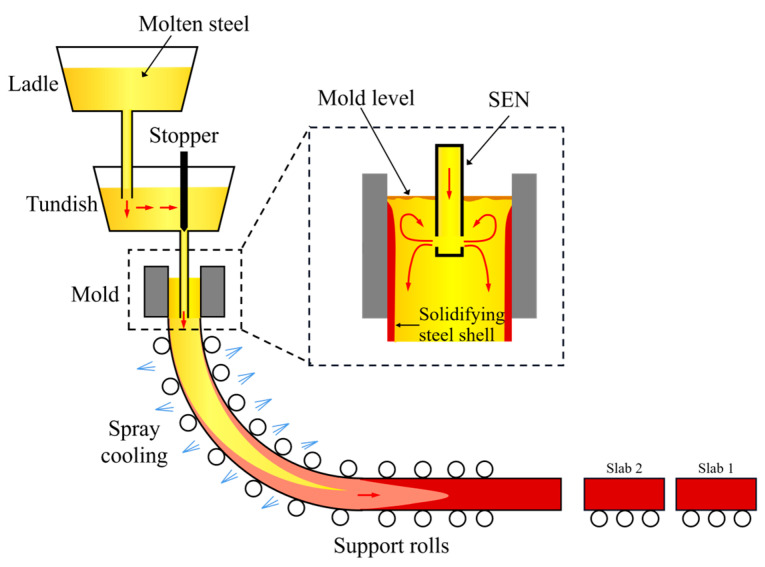
Schematic diagram of the continuous casting process.

The process flow of continuous casting is illustrated in Figure 1. In this process, liquid steel is first tapped into a ladle and then transferred to a tundish, which serves as a buffer and distributor. From there, the steel enters the mold through a submerged entry nozzle (SEN). Inside the mold, rapid heat extraction from water-cooled copper walls initiates shell formation. The complex flow pattern—highlighted by the recirculation loops within the mold—is crucial for temperature uniformity and inclusion flotation. Improper flow or inadequate mold control can lead to meniscus instability, shell thinning, and ultimately the formation of internal cracks. Downstream, the steel is guided through support rolls and spray zones in the secondary cooling area, where uneven cooling or strand bulging further increases crack susceptibility [28]. Therefore, collecting and analyzing process parameters across these regions is vital for crack prediction. A total of 981 process records were collected, representing sequential HSLA steel slab productions over a one-month period. Each record corresponds to a single cast billet and contains 70 process parameters, encompassing upstream ladle and tundish conditions, mold oscillation settings, cooling rates, and control variables from the secondary cooling zone.

The raw continuous casting process data inevitably contain noise, outliers, and missing values due to sensor faults, transmission errors, and non-standard operating conditions. Directly training on such noisy data may impair model performance or introduce biases. Therefore, we implemented a multi-step cleaning procedure.

First, a format and completeness check are performed to identify missing or null values. Records containing incomplete entries for critical variables (e.g., casting speed, mold level, and secondary cooling flow rate) were removed to avoid unintended biases or errors during model training.

Second, a business logic validation step was applied. This involved verifying the semantic correctness of each record based on operational knowledge. For example, casting speed must be positive, mold level values must fall within typical machine operating limits, and water flow or gas pressure values must not be zero under active production. Records that violated fundamental process logic were considered invalid and removed.

Third, statistical outlier detection was conducted using the 3-sigma criterion. For each continuous variable, values outside the range [μ−3σ,μ+3σ] were flagged as potential outliers, where μ and σ represent the sample mean and standard deviation, respectively. This method was applied to both steady-state parameters (e.g., average mold temperature) and time-varying indicators (e.g., casting speed deviation). Only those records that violated multiple checks (e.g., both logic and statistical rules) were discarded in order to preserve rare but valid operating conditions.

This multi-step procedure ensures the resulting dataset is both clean and representative of real industrial variability, providing a reliable foundation for feature selection, model training, and validation. After data cleaning, 733 valid samples remained, each containing 70 input variables and an associated internal crack label.

### 2.2. Feature Selection

Effective selection of relevant process parameters is crucial for improving prediction accuracy and reducing computational burden, especially when working with industrial datasets that contain a large number of variables [29]. In this study, we initially collected 70 process-related variables for each billet, such as casting speed, mold level, gas pressure, and secondary cooling flow. These variables are referred to as input features in the modeling process.

To identify which of these features are most relevant to defect formation, we applied a statistical method called mutual information (MI) [30]. MI measures the strength of association between each process parameter and whether or not the billet contains an internal crack. This crack status is recorded as a binary target label, where 1 means the billet has a crack and 0 means it does not.

Unlike linear correlation, MI captures both linear and nonlinear dependencies, making it suited for describing complex, nonlinear relationships often present in steel casting processes. For each candidate feature $X_{i}$, its mutual information with the target label *Y* (e.g., ground-truth classification label) was computed as follows [31]:

(1) $$I(X_{i};Y)=\sum_{x_{i}\in X_{i}}\sum_{y\in Y}p(x_{i},y)\log\left(\frac{p(x_{i},y)}{p(x_{i})p(y)}\right)$$

where $p(x_{i},y)$, $p(x_{i})$, and p(y) are the joint and marginal probability distributions estimated from the data. Features with low mutual information were considered redundant or irrelevant and subsequently excluded from model training.

**Figure 2:**
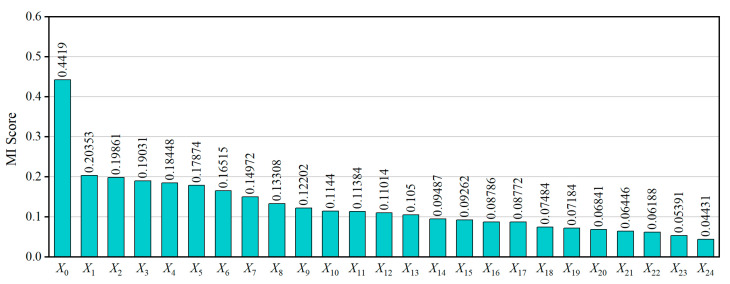
Mutual information score plot.

All features were evaluated using the MI criterion, and the top 25 were selected for subsequent model development. Figure 2 presents the MI scores of the top 25 most informative features, while Table 2 provides the physical descriptions of these selected variables. These features were used as input for all subsequent machine learning models. The Mn/S ratio (*X*_0_) shows the highest MI score (0.4419), indicating that chemical composition plays a dominant role in crack formation. Other top features include mold surface temperatures (*X*_1_ and *X*_3_), gas pressures (*X*_2_, *X*_7_, and *X*_8_), and cooling water flow rates (*X*_5_, *X*_6_, *X*_10_, and *X*_14_), all of which affect heat transfer and solidification stability. Oscillation parameters and drive forces also appeared, reflecting the combined thermal and mechanical effects on defect formation. The selected features cover a wide range of process domains, including upstream chemical indices, mold thermomechanical conditions, SEN injection dynamics, and secondary cooling control, thereby providing a comprehensive representation of the casting system.

## 3. Methodology

### 3.1. The Proposed Data Augmentation Strategy

**Figure 3:**
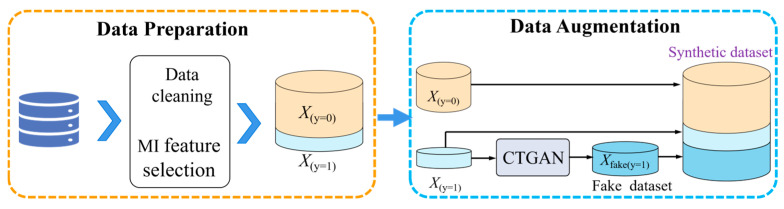
The proposed data augmentation strategy.

To address the issue of class imbalance inherent in the continuous casting process dataset, we propose a targeted data augmentation strategy, as illustrated in Figure 3. The original dataset is first subjected to preprocessing and feature selection, resulting in a cleaned dataset *X*. The majority class samples *X*_(*y*=0)_ are retained, while the minority class *X*_(*y*=1)_ remains severely underrepresented. To balance the class distribution, the Conditional Tabular Generative Adversarial Network (CTGAN) is employed to synthesize additional minority class samples *X_fake_*_(*y*=1)_. The generator is trained on the minority class samples and conditioned on relevant categorical and continuous features. Once trained, the generator produces realistic synthetic samples that are subsequently combined with the original data to form an augmented, balanced dataset.

This augmented dataset serves as the input for downstream model training, enhancing the robustness of the classifier under imbalanced conditions.

### 3.2. Conditional Tabular Generative Adversarial Network

**Figure 4:**
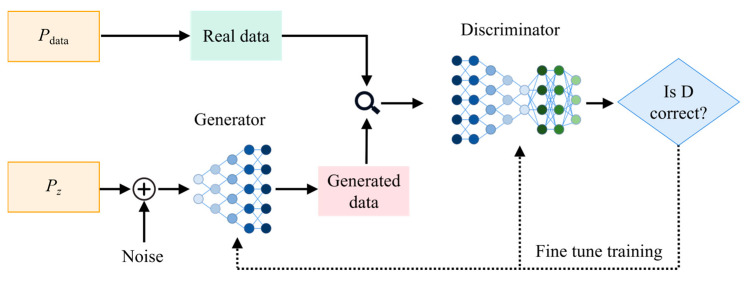
The architecture of GAN.

CTGAN is a GAN-based model specifically designed for synthesizing realistic tabular data, which often contain a mix of numerical and categorical variables. The architecture of CTGAN follows the standard GAN framework, which consists of a generator *G* and a discriminator *D*, as shown in Figure 4. The training process of CTGAN involves sampling real data from distribution $p_{\mathrm{data}}$ and random noise z from prior distribution $p_{z}(z)$. The generator learns to map random noise $z∼p_{z}(z)$ and conditional information *c* to synthetic data samples $\hat{x}$:

(2) $$\hat{x}=G(z,c)$$

The discriminator aims to distinguish between real data $x∼p_{\mathrm{data}}(x)$ and synthetic samples $\hat{x}$, optimizing the following adversarial loss:

(3) $$\underset{G}{\min}\underset{D}{\max}E_{x∼p_{\mathrm{data}}(x)}[\log D(x,c)]+E_{z∼p_{z}(z)}[\log(1-D(G(z,c),c))]$$

A key innovation of CTGAN is its conditional sampling strategy, where *c* is sampled from the discrete variables in the dataset. This conditioning enables the generator to learn class-specific and category-aware data distributions, which is essential for effectively balancing imbalanced datasets. Moreover, for continuous variables, CTGAN applies a variational Gaussian mixture model transformation to capture multimodal and skewed distributions. Given a continuous variable $x_{c}$, its transformed representation ${\tilde{x}}_{c}$ is modeled as follows:

(4) $$p({\tilde{x}}_{c})=\sum_{k=1}^{K}\pi_{k}N(\mu_{k},\sigma_{k}^{2})$$

where $p({\tilde{x}}_{c})$ is the conditional probability density of the transformed continuous variable ${\tilde{x}}_{c}$, $\pi_{k}$ is the mixture weight of the *k*-th Gaussian component, and $N(\mu_{k},\sigma_{k}^{2})$ denotes a normal distribution with mean $\mu_{k}$, and variance $\sigma_{k}$. This transformation stabilizes GAN training and improves the fidelity of generated continuous features.

In this study, the CTGAN model was trained using the original dataset, with the generator subsequently used to synthesize additional minority class samples.

### 3.3. Machine Learning Algorithms

CatBoost is a gradient boosting algorithm developed by Prokhorenkova et al. [32], designed to address key limitations of traditional Gradient Boosting Decision Trees (GBDTs), such as prediction shift and overfitting, particularly when dealing with categorical features and small datasets. Its primary innovations include ordered boosting and the use of symmetric trees, both of which contribute to improved stability and generalization. In this work, CatBoost is employed for its robust handling of categorical variables and its effectiveness in scenarios with limited training samples.

Given a training dataset $D={\left\{(x_{i},y_{i})\right\}}_{i=1}^{n}$, the CatBoost model constructs an ensemble of decision trees in an additive manner:

(5) $$F(x)=\sum_{t=1}^{T}\eta \cdot f_{t}(x)$$

where $f_{t}(x)$ is the output of the *t*-th tree, and *η* is the learning rate.

For multi-class classification with *C* classes, the predicted probability of instance $x_{i}$ belonging to class *c* is given by the softmax function:

(6) $$P(y_{i}=c|x_{i})=\frac{\exp(F_{c}(x_{i}))}{\sum_{k=1}^{C}\exp(F_{k}(x_{i}))}$$

The model is trained by minimizing the multi-class logarithmic loss (cross-entropy):

(7) $$L=-\sum_{i=1}^{n}\log P(y_{i}=y_{i}^{*}|x_{i})$$

where $y_{i}^{*}$ is the true class label.

To control overfitting, CatBoost introduces L2 regularization on the leaf values of each tree:

(8) $$L_{\mathrm{reg}}=L+\lambda \sum_{t=1}^{T}\sum_{j=1}^{L_{t}}w_{tj}^{2}$$

where *λ* is the regularization parameter, $L_{t}$ is the number of leaf nodes in tree *t*, and $w_{ij}$ is the value of the *j*-th leaf.

To improve the model’s performance, key hyperparameters such as learning rate, tree depth, and number of iterations were optimized using the particle swarm optimization (PSO) [33] algorithm. PSO efficiently explores the search space by simulating the collective behavior of particles, leading to parameter settings that enhance classification accuracy while avoiding manual tuning.

## 4. Results and Discussions

The proposed framework was implemented in PyTorch (version 2.4.1) and executed on a Linux-based server equipped with an Intel (R) Xeon (R) CPU E5-2640 @ 2.50 GHz and an NVIDIA 2080Ti GPU. The hyperparameters shown in Table 3 were optimized using PSO. The PSO configuration, including the number of particles, inertia weight, and acceleration constants. These values enable efficient exploration of the search space. The final CatBoost hyperparameters were chosen based on five-fold cross-validation results on the training set.

### 4.1. The Evaluation of the Synthetic Dataset

To evaluate the quality and effectiveness of the CTGAN-generated data, we conducted a comprehensive analysis from three perspectives: class distribution, feature-wise statistical alignment, and structural consistency in feature space. This section presents the results of these evaluations.

**Figure 5:**
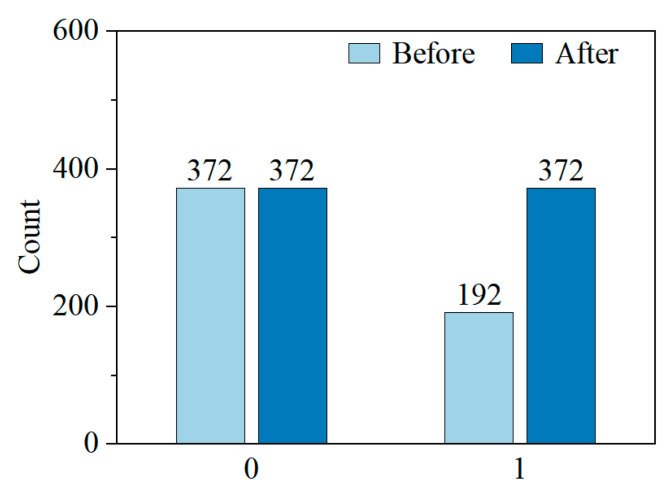
Class distribution before and after CTGAN-based augmentation.

Figure 5 shows the label distribution in the training set before and after CTGAN augmentation. Initially, the dataset was imbalanced, with 372 normal samples (label 0) and only 192 cracked samples (label 1). After augmentation, both classes contain 372 samples, achieving a fully balanced dataset. This adjustment addresses the class imbalance issue that often hinders model performance, especially in identifying minority class instances. Unlike traditional oversampling, CTGAN generates synthetic samples by capturing the joint distribution of features, preserving complex relationships within the minority class. These results demonstrate the effectiveness of CTGAN in creating a balanced and realistic dataset, providing a reliable foundation for model training in the subsequent analysis.

**Figure 6:**
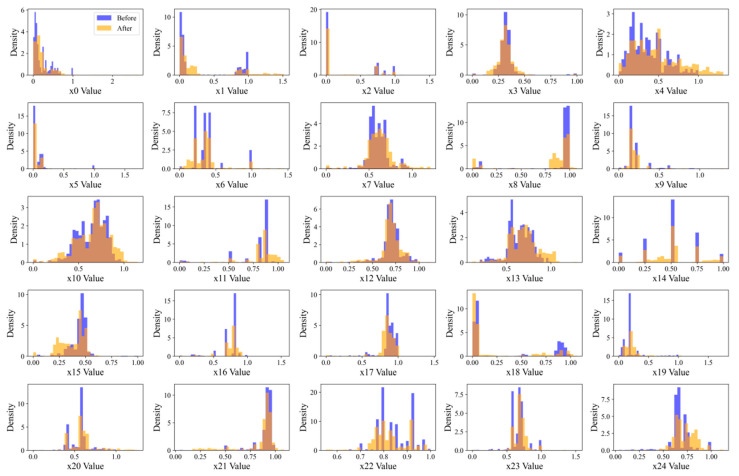
Comparison of feature distributions before and after data augmentation.

Figure 6 presents the distributional comparison of 25 selected features in the minority class before and after CTGAN augmentation. The original data are shown in blue, while the CTGAN-generated samples are depicted in orange. Geometrically, the distributions of most features remain well aligned, with synthetic samples closely following the original ones in terms of density peaks, spread, and modality. For instance, features such as *X*_3_, *X*_6_, *X*_10_, *X*_13_, and *X*_23_ display similar shapes and ranges, suggesting that the generator is able to approximate the marginal distributions of these features with reasonable accuracy. Some features, including *X*_0_, *X*_14_, and *X*_17_, show a sharper mode shift in the generated data, which might slightly affect their contribution to classification decision boundaries. Despite these minor discrepancies, the overall augmentation result preserves the essential geometric and statistical properties of the original feature space. This distributional consistency helps maintain the integrity of the minority class structure while mitigating sample imbalance. This helps mitigate class imbalance while preserving the statistical integrity necessary for reliable model generalization.

**Figure 7:**
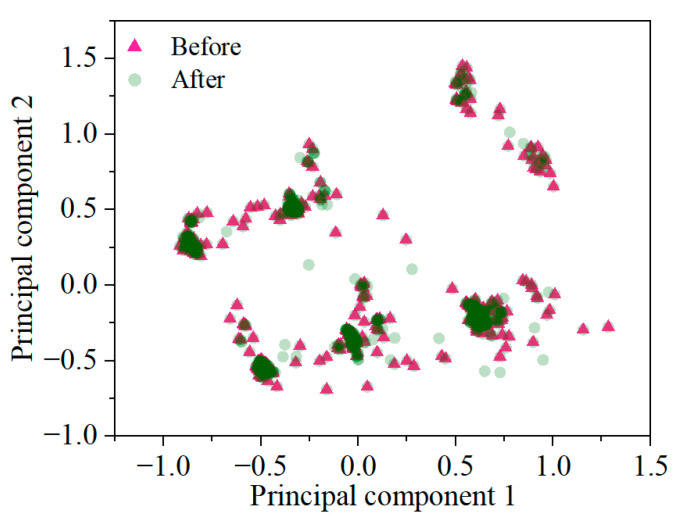
Visualization of synthetic sample distributions using PCA.

To further examine the structural fidelity of the generated samples, a PCA projection was conducted. PCA was used to project high-dimensional features into a two-dimensional space to visually assess the structural similarity between original and augmented data. As shown in Figure 7, the synthetic samples (green circles) generated by CTGAN closely align with the original minority class samples (magenta triangles) in the principal component space. Most synthetic points are located within or near the original clusters, indicating that the generator successfully captures the dominant feature structure. A few dispersed samples appear in low-density regions, suggesting added diversity without significant distributional shift. The PCA projection confirms that the augmented data preserves the global structure of the original class, supporting its suitability for model training.

### 4.2. Model Prediction Results

In order to evaluate the performance of the binary classification model developed in this study, several widely used metrics were adopted, including Accuracy, Precision, Recall, and F1 Score. These metrics are computed based on the confusion matrix, which consists of true positives (TPs), true negatives (TNs), false positives (FPs), and false negatives (FNs). Their definitions are as follows:

(9) $$\mathrm{Accuracy}=\frac{\mathrm{TP}+\mathrm{TN}}{\mathrm{TP}+\mathrm{FP}+\mathrm{TN}+\mathrm{FN}}$$

(10) $$\mathrm{Precision}=\frac{\mathrm{TP}}{\mathrm{TP}+\mathrm{FP}}$$

(11) $$\mathrm{Recall}=\frac{\mathrm{TP}}{\mathrm{TP}+\mathrm{FN}}$$

(12) $$F1=2\times \frac{\mathrm{Precision}\times \mathrm{Recall}}{\mathrm{Precision}+\mathrm{Recall}}$$

Accuracy reflects the overall correctness of predictions. Precision measures the proportion of true positive predictions among all positive predictions. Recall quantifies the proportion of actual positive samples correctly identified. The F1 Score provides a balanced measure of Precision and Recall, particularly useful in imbalanced datasets.

**Figure 8:**
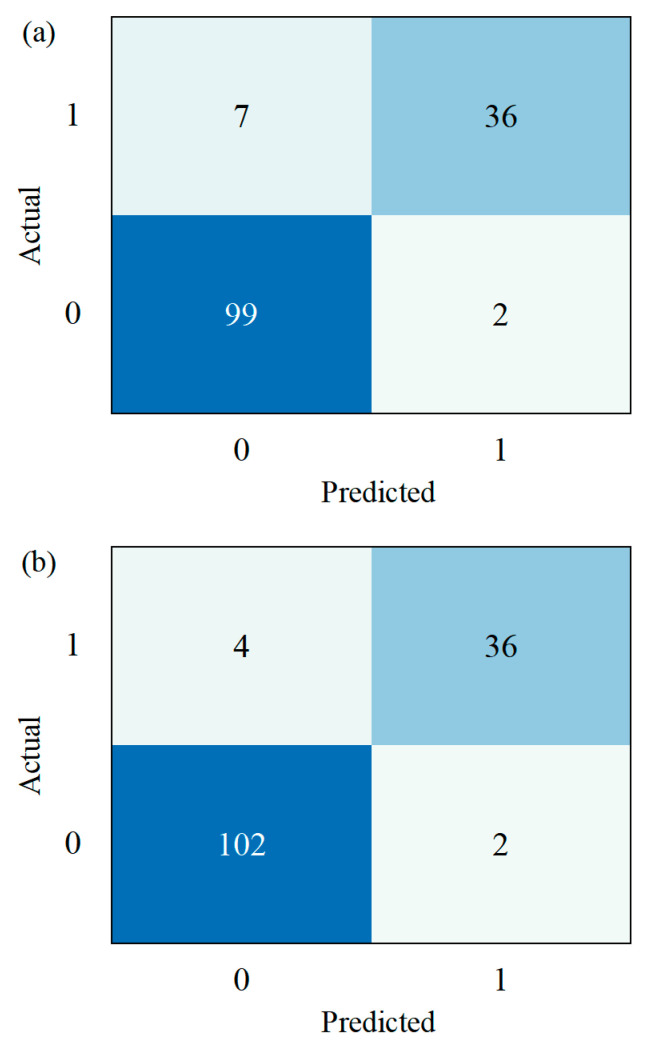
Confusion matrix comparisons for the model trained with different datasets: (**a**) original dataset; (**b**) dataset with CTGAN augmentation.

**Figure 9:**
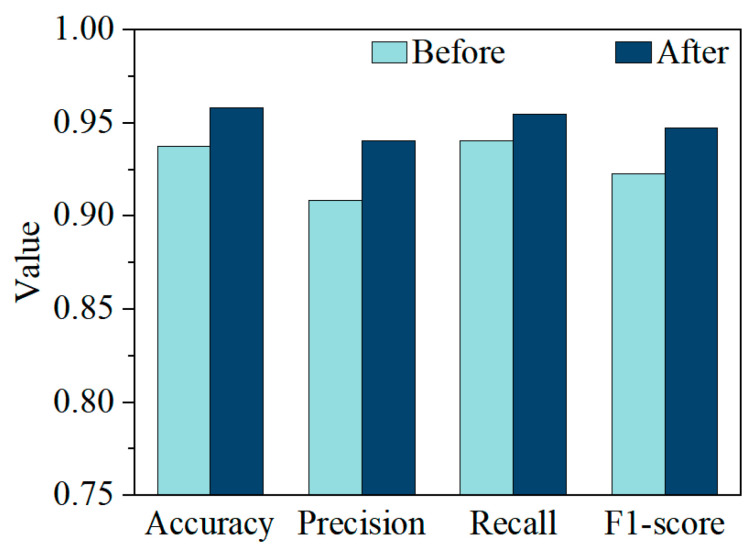
Evaluation metrics of the model before and after data augmentation.

Figure 8 shows the confusion matrices of the classification model before and after CTGAN augmentation. The diagonal entries represent correctly predicted samples, while the off-diagonal entries indicate misclassifications. After CTGAN augmentation, the model has 3 fewer missed detections for the presence of internal cracks. Figure 9 compares the classification performance before and after CTGAN-based data augmentation across four metrics: accuracy, precision, recall, and F1-score. Overall, all metrics improved after augmentation, indicating enhanced model effectiveness. Accuracy increased slightly, suggesting overall prediction correctness improved. Precision and recall both showed noticeable gains, with recall improving the most, which reflects a stronger ability to detect minority class (crack) samples. Consequently, the F1-score also increased, indicating a better balance between precision and recall. These results confirm that CTGAN effectively alleviates class imbalance, enabling the classifier to better recognize defect samples without sacrificing overall performance.

### 4.3. Comparison with Other Models

To evaluate the effectiveness of CTGAN-based data augmentation in improving defect prediction, we conducted comparative experiments using different machine learning models, including KNN, SVM, and MLP. KNN is a simple architecture that enables classification using sample distance metrics. SVM is a kernel-based hyperplane optimization that enables strong generalization. MLP is a neural network model that enables nonlinear pattern learning. These models, extensively used in classification tasks due to their performance, interpretability, and application, function as benchmarks for comparison study with the proposed CatBoost.

All experiments were conducted using five-fold cross-validation to ensure the robustness and generalizability of the results. Table 4 summarizes the performance of the four models on both the original imbalanced dataset and the CTGAN-augmented dataset. Among all models, CatBoost consistently achieved the best results. Its F1-score increased from 0.8927 on the original dataset to 0.9022 after augmentation, while accuracy improved from 0.9169 to 0.9239. KNN and MLP also showed performance gains, particularly in recall and F1-score, indicating enhanced sensitivity to minority class (crack) instances after augmentation. SVM, however, exhibited only marginal improvements, suggesting limited benefit from the synthetic data. It is worth noting that the precision of the MLP model decreased from 0.9015 to 0.8271 after CTGAN augmentation, even though recall and F1-score improved. This may be due to the MLP’s higher sensitivity to synthetic sample variability. The neural network may interpret some augmented minority-class samples as valid signals, leading to an increase in false positives and, thus, lower precision. In contrast, tree-based models like CatBoost are more robust to such sample shifts due to their structure and regularization mechanisms. These results demonstrate that CTGAN-based data augmentation improves minority class detection without compromising overall model accuracy. CatBoost, in particular, showed superior and stable performance across all metrics, making it a reliable choice for downstream defect prediction tasks.

### 4.4. Machine Learning Explanation with SHAP

In industrial applications such as steel production, interpreting the internal logic of machine learning models is crucial for improving process transparency and trustworthiness. SHAP offers an effective framework for assessing how individual features influence model outputs, capturing both isolated and combined effects to provide detailed interpretability. Rooted in the Shapley value theory from cooperative game theory, SHAP quantifies how much each feature contributes—either positively or negatively—to a given prediction. By aggregating these contributions across the dataset, it enables a comprehensive evaluation of global feature importance.

**Figure 10:**
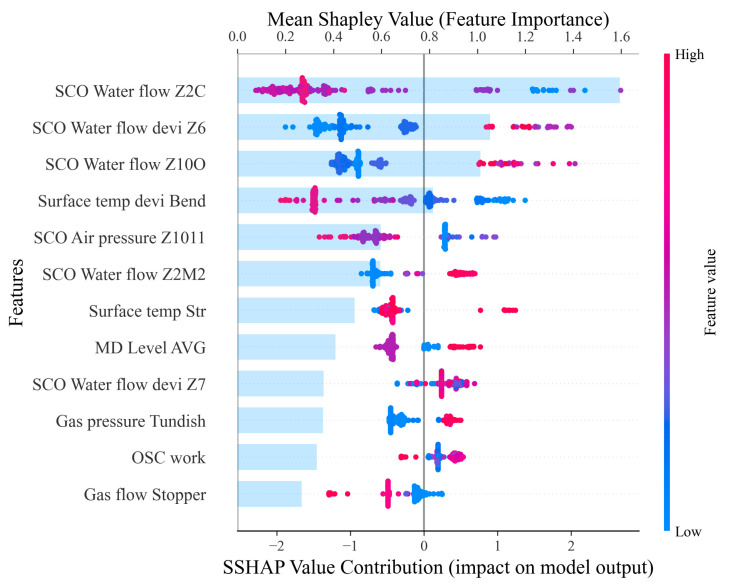
SHAP summary plot of the top 12 process features and their contributions to crack prediction.

The SHAP values were calculated using the SHAP package in Python (version 3.8.13), and their overall importance and summary distribution are illustrated in Figure 10 through the summary_plot function. In Figure 10, the upper horizontal axis denotes the mean SHAP value, indicating the global importance of each feature across all samples. The lower axis represents the SHAP values for individual instances, reflecting the direction and magnitude of each feature’s contribution to the model prediction. Each row corresponds to a specific feature. The light blue bars indicate the average SHAP value, with longer bars denoting higher overall importance. The colored scatter points represent individual samples, where the red points correspond to positive SHAP values, reflecting a feature’s positive influence on the predicted label, whereas blue points represent negative SHAP values, indicating a suppressing effect.

To avoid redundancy and emphasize key drivers, the analysis is restricted to the top 12 features for quantitative and visual interpretation. As demonstrated in Figure 10, the most influential features are related to secondary cooling zone water flow rates and their deviations, such as Z2C, Z6, Z10O, and Z2M2. High values in these features (red dots on the right) substantially increase the predicted crack probability, suggesting that excessive localized cooling may cause thermal stress concentration and solidification cracking. It should be noted that while SHAP explains model predictions, further metallurgical validation is required to confirm the causal relationships between high water flow rates and crack formation. These findings suggest that uneven or excessive secondary cooling in specific zones may be linked to increased crack risks, indicating the need for optimized water distribution strategies. Other features such as SCO Air pressure Z1011, SCO Water flow Z2M2, and Surface temp Str also show a tendency where higher values correspond to positive contributions to the predicted output. In summary, features related to secondary cooling water flow, surface temperature fluctuations, and gas pressure exhibit strong influence on the predicted crack risk and should be prioritized during process optimization and monitoring.

## 5. Conclusions

This study presents a data-driven framework for predicting internal cracks in HSLA steel billets during continuous casting, combining CTGAN for data augmentation and CatBoost for classification. The model is developed and evaluated using real process data collected from a full-scale industrial continuous casting line. A total of 70 casting parameters were initially extracted, from which 25 informative features were selected based on mutual information analysis. These variables include mold level, secondary cooling flow, and gas injection pressure. To address the issue of class imbalance, CTGAN is employed to generate synthetic minority samples that preserve the distributional structure of real defect cases. CatBoost is then trained on the augmented dataset and benchmarked against other classifiers, including KNN, SVM, and MLP. Experimental results demonstrate that the proposed CTGAN–CatBoost achieved the highest performance, with its F1-score increasing from 0.8927 to 0.9022 and accuracy from 0.9169 to 0.9239 after augmentation.

Nevertheless, the proposed model is subject to certain limitations. The dataset used in this study was collected from a single production line and covers a limited range of casting conditions. Since CTGAN learns from empirical feature distributions, the performance of both the generative model and the classifier is closely tied to the characteristics of the training data. As such, applying the current model to other casting machines or production environments would require retraining with data representative of those conditions. Future study will focus on expanding the dataset to multiple production lines and casting conditions, integrating real-time process data, and incorporating domain knowledge to enhance model generalizability and industrial applicability.

## Figures and Tables

**Table 1:** Experimental casting machine equipment parameters.

Parameter	Unit	Value/Type
Metallurgical length	m	35.1
Mold height	mm	900
Mold width	mm	870–1977
Casting speed	m/min	0.8–1.5
Lubrication method	-	Mold Flux
Furnace capacity	ton	200
Caster radius	m	9.1
Slab thickness	mm	230–250

**Table 2:** Description of selected top 25 features.

No.	Parameters	Unit	Description
*X*_0_	Mn-S ratio	%	Manganese-to-sulfur ratio index in steel
*X*_1_	Surface temp Str edge	°C	Straightener segment edge surface temperature
*X*_2_	Gas pressure tundish	%	Gas pressure at the submerged entry nozzle tip
*X*_3_	Surface temp bend	bar	Bender segment edge surface temperature
*X*_4_	MD level AVG	mm	Width of continuous casting mold
*X*_5_	SCO water flow Z2M2	L/min	Secondary cooling zone Z2M2 water flow rate
*X*_6_	SCO water flow Z2C	L/min	Secondary cooling zone Z2C water flow rate
*X*_7_	SEN nozzle gas pressure	bar	Gas pressure in the submerged entry nozzle body
*X*_8_	Gas pressure SEN	bar	Gate sealing gas pressure in the ladle/tundish/SEN.
*X*_9_	OSC work	J	Oscillation energy consumption per cycle
*X*_10_	SCO water flow Z1NR	L/min	Secondary cooling zone Z1NR water flow rate
*X*_11_	OSC frequency	1/min	Mold oscillation frequency
*X*_12_	Casting speed cooling	m/min	Minimum casting speed at cooling segment
*X*_13_	Surface temp devi bend	°C	Bender segment surface temperature deviation
*X*_14_	SCO water flow Z10O	L/min	Secondary cooling zone Z10O water flow rate
*X*_15_	Drive force Bend AVG	N	Average bending segment drive force
*X*_16_	SCO air pressure Z1011	bar	Secondary cooling zone Z1011 air pressure
*X*_17_	TD inflow rate	ton/min	Tundish steel inflow rate
*X*_18_	MCO water temp devi WF	°C	Mold cooling water temperature difference in WF loop
*X*_19_	Gas flow stopper	L/min	Stopper/gate inert gas flow rate
*X*_20_	SCO water flow devi Z7	L/min	Secondary cooling zone Z7 water flow deviation
*X*_21_	Surface temp Str	°C	Straightener segment surface temperature
*X*_22_	Drive force Str AVG	N	Average straightener segment drive force
*X*_23_	SCO water flow devi Z6	L/min	Secondary cooling zone Z6 water flow deviation
*X*_24_	Steel weight tundish	ton	Steel weight in tundish

**Table 3:** Parameter setting of CatBoost.

Parameters	Value
Initial particle number	20
Max iterations of PSO	50
Inertia weight	0.8
Acceleration constant c1/c2	1.5, 1.5
Iterations	800
Learning rate	0.3
Depth	5
L2 regularization	0.6

**Table 4:** Predictive performance of different models.

Method	Original Dataset				CTGAN			
	Acc	Pre	Rec	F1	Acc	Pre	Rec	F1
CatBoost	0.9169	0.8951	0.8920	0.8927	0.9239	0.9041	0.9018	0.9022
KNN	0.8699	0.8372	0.8231	0.8284	0.8880	0.8570	0.8555	0.8557
SVM	0.8131	0.7677	0.7662	0.7593	0.8145	0.7692	0.7704	0.7622
MLP	0.8519	0.9015	0.8211	0.8140	0.8644	0.8271	0.8297	0.8275

## Data Availability

The original contributions presented in the study are included in the article, further inquiries can be directed to the corresponding author.
